# Trim28 Contributes to EMT via Regulation of E-Cadherin and N-Cadherin in Lung Cancer Cell Lines

**DOI:** 10.1371/journal.pone.0101040

**Published:** 2014-07-01

**Authors:** Lu Chen, Teresita Muñoz-Antonia, W. Douglas Cress

**Affiliations:** Molecular Oncology Program, H. Lee Moffitt Cancer Center and Research Institute, Tampa, FL, United States of America; Ghent University, Belgium

## Abstract

In previous work, we demonstrated that transcription factor Trim28 (Tripartite motif containing 28) plays a tumor-suppressor role in early-staged adenocarcinoma of the lung due to its ability to restrain transcription of cell cycle-regulating genes. Herein we examine Trim28's role in the epithelial-to-mesenchymal transition (EMT) which is strongly implicated in cancer metastasis. We found that Trim28 plays a role in TGF-β-induced EMT in non-small cell lung cancer cells. Silencing Trim28 with inhibitory RNAs alters the expression of numerous EMT markers, such as E-cadherin and N-cadherin, whereas overexpression of Trim28 has an opposite effect. Trim28 expression is induced following TGF-β treatment at both protein and mRNA levels. Trim28 deficiency impairs TGF-β-induced EMT and decreases cell migration and invasion. Finally, we demonstrate that the expression of Trim28 affects the acetylation and methylation of histones on E-cadherin and N-cadherin promoters. These results suggest that Trim28 contributes to EMT and might be important for tumor metastasis in lung cancer. Taken together with our previous work these results suggest a model in which Trim28 is a tumor suppressor early in the transformation process in lung cancer, but in later stages it functions as an oncogene.

## Introduction

The epithelial-to-mesenchymal transition (EMT) is characterized by a loss of cell-cell adhesion and polarity, down-regulation of epithelial markers, and acquisition of mesenchymal markers and phenotype [1]. Accumulating evidence from investigations on EMT have implicated many signaling pathways, including TGF-β, Notch, Wnt, EGF and FGF [2]. Among these, TGF-β induces EMT efficiently in a variety of model cell lines and *in vivo* [3]. Like many other biological processes, EMT is ultimately regulated transcriptionally by a number of transcription factors such as Snail1, Snail2, ZEB1 and ZEB2 [4]. In addition, epigenetic modifiers have also been shown to play a role in the regulation of EMT. For examples, Histone deacetylase SirT1 induces EMT through transcription factor ZEB1 whereas histone methyltransferase SUV39H1 and G9a repress E-cadherin expression and induce EMT in a Snail-dependent manner [5], [6], [7].

Trim28 is member of an evolutionally conserved family of transcription co-factors that have diverse functions [8]; including the regulation of cell proliferation, DNA repair, differentiation and pluripotency [9], [10], [11], [12]. Trim28 has been specifically implicated in the activation of EMT in fibroblasts [13] and cervical cancer cells [14]. Dependent upon context, Trim28 can either activate or repress transcription through distinct mechanisms [15], [16], [17], [18]. Trim28-mediated gene silencing has been shown to involve an association with histone methyltransferase SETDB1, histone deacetylase complex NuRD and recruitment to promoter regions via sequence-specific transcription factors [19], [20]. This complex suppresses target gene transcription by altering the histone modifications on promoters [8]. When phosphorylated at S824, Trim28 has been shown to associate with transcription factor Oct3/4 and activate Oct3/4 target genes transcription [12]. Another mechanism of Trim28-mediated transcription activation is via recruitment to specific response elements, such as glucocorticoid-responsive element (GRE) and Nur response element (NuRE), by NGFI-B and C/EBPβ respectively [17],[18]. Recent studies have suggested that Trim28 might play a role in cancer development and metastasis [13], [14]. Specifically, Trim28 expression is up-regulated in cancer tissues compared to normal tissues [9], [21], [22], [23]. In a search for breast cancer metastasis-associated proteins, Trim28 was identified as one of 197 proteins with elevated expression in metastatic tissues [24].

In our previous work [9], we discovered that high Trim28 (Tripartite motif containing 28) proteins levels correlate with longer overall survival in early-staged lung adenocarcinoma patients, but not in late-staged patients. This observation suggested to us that Trim28 might play dual roles in lung cancer. We speculated that the role in later stages might relate to EMT [14]. In order to test this hypothesis, we examined the effect of shRNAi-mediated Trim28 depletion on EMT in NSCLC cell lines (A549 and H358). We found that Trim28 contributes to TGF-β-induced EMT, and that high Trim28 favors EMT suggesting that Trim28 might be a regulator of tumor metastasis, particularly in the later stage of lung cancer development.

## Materials and Methods

### Cell lines, plasmids, and reagents

Original lung cancer cell lines were obtained from the ATCC. A549 and H358 cells were grown in RPMI 1640 with 10% fetal bovine serum. Trim28-deficient A549 cells (Trim28 shRNA), Trim28-proficient A549 and similarly derived control cells lines were described previously [9]. H358 cells stably expressing Trim28 shRNA were selected in 1 µg/ml puromycin. H358 cells stably expressing Trim28 were selected in 500 µg/ml G418. Single clones were screened for Trim28 expression. Similarly-derived control cell lines were generated using empty vectors. PcDNA-FLAG-HA-TIF1β and pRL-TK were described previously [9]. pCS2-Myc-Trim28 [25] and pGL3-E-cadherin [26] were the generous gifts from Ryan Potts (UT Southwestern) and Jia Fang (Moffitt Cancer Center), respectively. TGF-β was purchased from R&D Systems (240-B-002).

### Protein Analysis

Westerns were performed as previously described [27]. E-cadherin (#3195S), N-cadherin (#4061S), and β-tubulin (#2128S) antibodies were purchased from Cell Signaling Technology. Trim28 antibody (A300-275A) was from Bethyl Laboratories. Flag (F3165), β-actin (A5441), and γ-tubulin (T6557) antibodies were from Sigma. Luciferase assays were performed as previously described [9]. Cells were harvested 48 hrs after transfection and luciferase activity was determined using the Dual-Luciferase Reporter Assay System (Promega) following the manufacturer's protocol. Experiments were carried out in biological triplicate and all were repeated three times. Renilla luciferase vector pRL-TK was used to control for transfection efficiency.

### Q-RT-Quantitative Real-time PCR

Total cell RNA was harvested using the RNeasy Mini Kit (Qiagen) following the manufacturer's instructions. Reverse transcription reactions were carried out using iScript cDNA Synthesis Kit (Bio-Rad). Real-time PCR was performed using PerfeCTa SYBR Green Supermix on a CFX96 real-time PCR detection system. The primers used are listed in Table 1.

**Table 1:** Primers used in Q-RT-Quantitative Real-time PCR and ChIP assay.

Target gene	Forward primer	Reverse primer	Method
Trim28	5′- tgtttccacctggactgtca -3′	5′- ccagcagtacacgctcacat -3′	Q-RT-PCR
Twist1	5′- tgcatgcattctcaagaggt -3′	5′- gttttgcaggccagtttgat -3′	Q-RT-PCR
Snail1	5′- gctccacaagcaccaagagt -3′	5′- attccatggcagtgagaagg -3′	Q-RT-PCR
Snail2	5′- ctttttcttgccctcactgc -3′	5′- acagcagccagattcctcat -3′	Q-RT-PCR
Vimentin	5′- gagaactttgccgttgaagc -3′	5′- tccagcagcttcctgtaggt -3′	Q-RT-PCR
E-cadherin	5′- caatgccgccatcgcttac -3′	5′- atgactcctgtgttcctgttaatg -3′	Q-RT-PCR
N-cadherin	5′- gacaatgcccctcaagtgtt -3′	5′- ccattaagccgagtgatggt -3′	Q-RT-PCR
Fibronectin	5′- accaacctacggatgactcg -3′	5′- gctcatcatctggccatttt -3′	Q-RT-PCR
GAPDH	5′- gagtcaacggatttggtcgt -3′	5′- ttgattttggagggatctcg -3′	Q-RT-PCR
E-cadherin	5′- ggccggcaggtgaaccctca -3′	5′- gggctggagtctgaactga -3′	ChIP assay
N-cadherin	5′- ggtgctcaagggctctactg -3′	5′- gtgagaggccagactcgttc -3′	ChIP assay

### Immunofluorescence Microscopy

Immunofluorescence microscopy was performed as previously described [9]. Primary antibodies used for staining were from Cell Signaling Technology (anti-E-cadherin #3195S and anti-β-tubulin #2128S). AlexaFluor 488-conjugated goat anti-rabbit (Invitrogen) was used as secondary antibody. Cells were imaged by the Microscopy Core at Moffitt Cancer Center using a Leica SP5 AOBS tandem scanning inverted confocal microscope.

### Chromatin immunoprecipitation assays

ChIP assays were performed as previously described [9]. Antibodies used for immunoprecipitation include normal rabbit IgG (12–370, Millipore), anti-acetyl-histone H3K9 (07–352, Millipore), anti-histone H3 trimethyl K9 (ab8898, Abcam) and anti-histone H3 dimethyl K9 (ab1220, Abcam). The precipitated DNA was purified using QIAquick PCR Purification Kit (Qiagen). Real-time PCR was performed using PerfeCTa SYBR Green SuperMix on a Bio-Rad CFX96 real-time PCR detection system. The primers used are listed in Table 1.

### Wound-healing assays

Cells were seeded in 6-well plates and cultured until >90% confluent. Three straight wounds were scratched in each well using a sterile 200 µl pipette tip. Cells were rinsed gently with PBS and 2 ml of media (without FBS or containing 10% FBS) added. Pictures were taken at 40x magnification right after scratching and again after 24 hrs and 48 hrs. All assays were performed in triplicate and all experiments repeated three times.

### Invasion assays

Invasion assays were performed in BD BioCoat Matrigel Invasion chamber (354480, BD Bioscences) following the manufacturer's instructions. Specifically, A549 cells were resuspended at 5×10^4^ cells/ml and loaded into chamber inserts (control inserts or Matrigel-coated inserts). Cells were incubated at 37°C for 22 hrs and the cells that had migrated were washed, fixed with methanol, stained with 0.5% crystal violet and counted in three microscopic fields. The percent invasion was calculated by dividing the mean number of cells invading through a Matrigel insert by the mean number of cells migrating through a control insert. The invasion index was determined by comparing the percent invasion of Trim28 knockdown cells with the percent invasion of control cells.

### Three-dimensional multicellular spheroid cultures

Three-dimensional multicellular spheroid cultures were created similarly as described [28]. Briefly, cells were resuspended in growth media to a concentration of 5×10^6^ cells/ml, 30 µl of the cell suspension were used to make a droplet and 12 droplets were loaded onto the lid of a sterile 10-cm tissue culture plate. The lid was carefully flipped and 20 µl of growth media was added to each droplet before being placed on a 10-cm tissue culture plate containing 6 ml of growth media and incubated overnight. The next day, 20 µl of media was removed from each droplet and 20 µl of fresh media without TGF-β or containing TGF-β (final concentration; 5 ng/ml) was added. Cells were incubated for 72 hrs and harvested for RNA extraction.

### Statistical analysis

Experiments were performed in triplicate and presented as the mean +/− SD. The data were analyzed by two-tailed Student's *t*-test for significance. *P* values less than 0.05 were considered statistically significant and represented by asterisks in figures.

## Results

### Trim28 deficiency alters the expression of EMT markers

**Figure 1:**
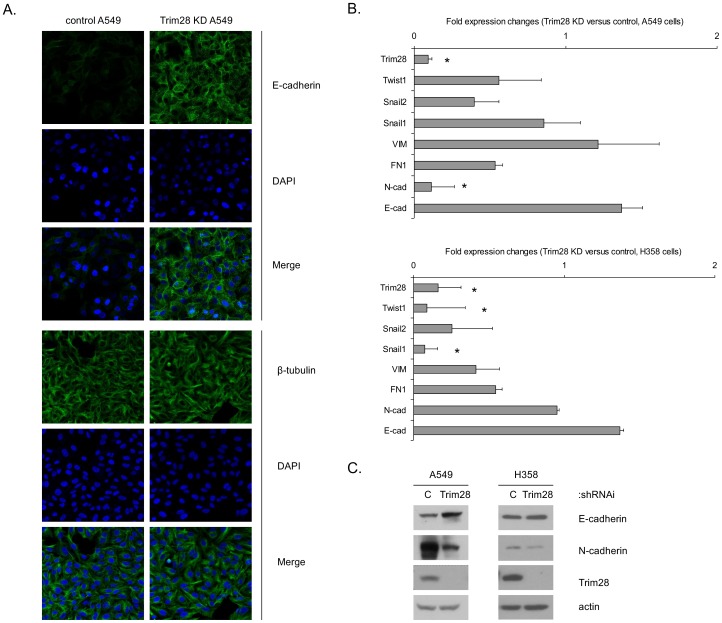
Trim28 deficiency alters the expression of EMT markers. *A*, control and Trim28 knockdown A549 cells were stained (as described under “Experimental Procedures”) and examined using confocal immunofluorescence microscopy, as follows: E-cadherin (green, top panel), β-tubulin (green, bottom panel), and DAPI (blue). Merged images are in the bottom of each panel. *B*, RNA extracts of control and Trim28 knockdown A549 and H358 cells were subjected to real-time PCR to determine the expression of target genes as indicated. *Asterisks* represent significant *p* values, *: *p*<0.05. *C*, the expression of EMT markers E-cadherin and N-cadherin were measured in control and Trim28 knockdown A549 and H358 cells by western blotting.

To address the role of Trim28 in A549 and H358 non-small cell lung cancer cell lines, control and Trim28 deficient cells were developed using non-silencing shRNA as a control and shRNA targeting Trim28. First, we utilized an E cadherin antibody and confocal microscopy to image E-cadherin expression in control A549 cells and in Trim28-deficient A549s. Fig. 1A demonstrates that control A549 cells express relatively low levels of E cadherin, whereas Trim28-deficient cells express a great deal of E cadherin localized in the plasma membrane. Levels of β-tubulin were unaffected by Trim28-deficiency. Real-time PCR was used to examine the expression of additional EMT markers in control and Trim28-deficient A549s and H358 cells. As shown in Fig. 1B, E-cadherin mRNA is increased while N-cadherin and Snail2 were decreased in Trim28 knockdown cells. In H358 cells (Fig. 1B bottom) E-cadherin is increased while Fibronectin, Snail1, Snail2, and Twist1 are decreased in Trim28 knockdown cells. Western blots confirmed that Trim28 knockdown resulted in an increase in E-cadherin expression and a reduction in N-cadherin expression at the protein level (Fig. 1C). The reduction in N-cadherin protein was also evident by confocal microscopy (see Fig S1). The respective positive correlations between Trim28 expression and N-cadherin expression and the negative correlation between Trim28 expression and E-cadherin expression are also evident when a series of unmanipulated cell lines are examined by Western blotting (see Fig S2).

### Trim28 overexpression alters the expression of EMT markers

**Figure 2:**
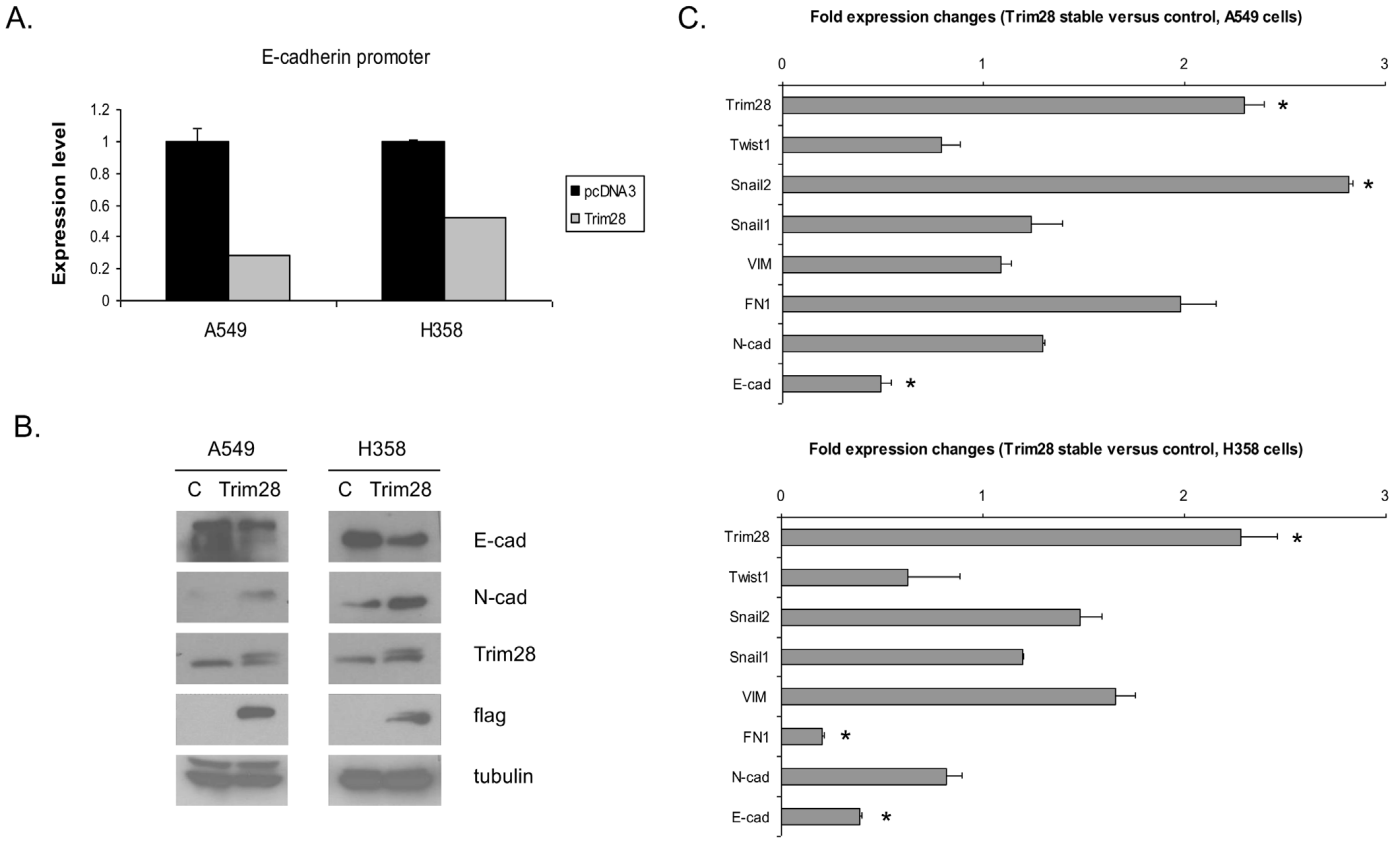
Trim28 proficiency alters the expression of EMT markers. *A*, A549 and H358 were transfected in triplicate with E-cadherin promoter luciferase reporter, pRL-TK reporter, and expression plasmids indicated in the figure. Cells were harvested 48 hrs after transfection for determination of luciferase levels. *B*, control (pcDNA3) and Flag-Trim28 stably expressed A549 and H358 cells were analyzed for E-cadherin and N-cadherin levels by western blotting. *C*, RNA extracts of control and Trim28 stably expressed A549 and H358 cells were subjected to real-time PCR. The expression of target genes was determined. *Asterisks* represent significant *p* values, *: *p*<0.05.

Since previous work has reported that Trim28 can act as a transcription co-factor [16], [17], we asked whether Trim28 can regulate the activity of the E-cadherin promoter using a luciferase reporter construct driven by the E-cadherin promoter. Empty pcDNA vector or pCS2-Myc-Trim28 plasmids were transfected into A549 and H358 cells together with an E-cadherin promoter-driven luciferase reporter and a transfection control reporter (pRL-TK Renilla Luciferase). Fig. 2A shows that the E-cadherin promoter is significantly repressed by Trim28 overexpression in both cell lines. To address a greater number of markers A549 and H358 cells stably expressing Trim28 were derived along with control lines similarly-derived using empty vector. As expected, Trim28 overexpression resulted in reduced E-cadherin expression levels whereas N-cadherin levels were increased (Fig. 2B). Similar results (Fig. 2C) were obtained at the mRNA level utilizing real-time PCR. Collectively, these data suggest that Trim28 can promote EMT in lung cancer cell lines.

### Trim28 expression is induced by TGF-β

**Figure 3:**
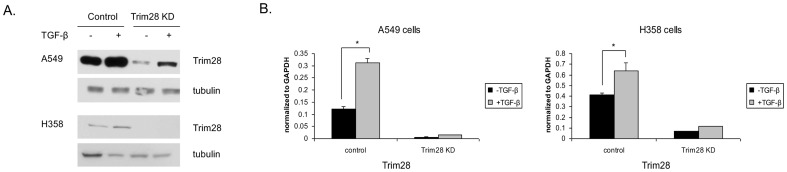
Trim28 expression is induced by TGF-β. *A* and *B*, A549 and H358 stably expressing non-silencing shRNA and Trim28 shRNA were treated with TGF-β (2 ng/ml) for 3 to 10 days. Whole cell lysates and RNA extracts were subjected to western blotting using the indicated antibodies (A) and real-time PCR (B). *Asterisks* represent significant *p* values, *: *p*<0.05.

It is known that Trim28 expression and TGF-β signaling are both increased in a variety of cancers including lung [9], [23], [29]. To determine if these observations are connected in lung cancer, we utilized two non-small cell lung cancer cell lines that undergo EMT upon TGF-β treatment as models [30], [31]. As can be seen in Figure 3A, Trim28 protein levels are increased upon TGF-β treatment in both cell lines. Although the basal level of Trim28 was very low in Trim28 knockdown cells, its expression was nonetheless induced following TGF-β incubation. Real-time PCR showed the similar result in mRNA levels (Figure 3B).

### Trim28 participates in TGF-β-induced EMT

**Figure 4:**
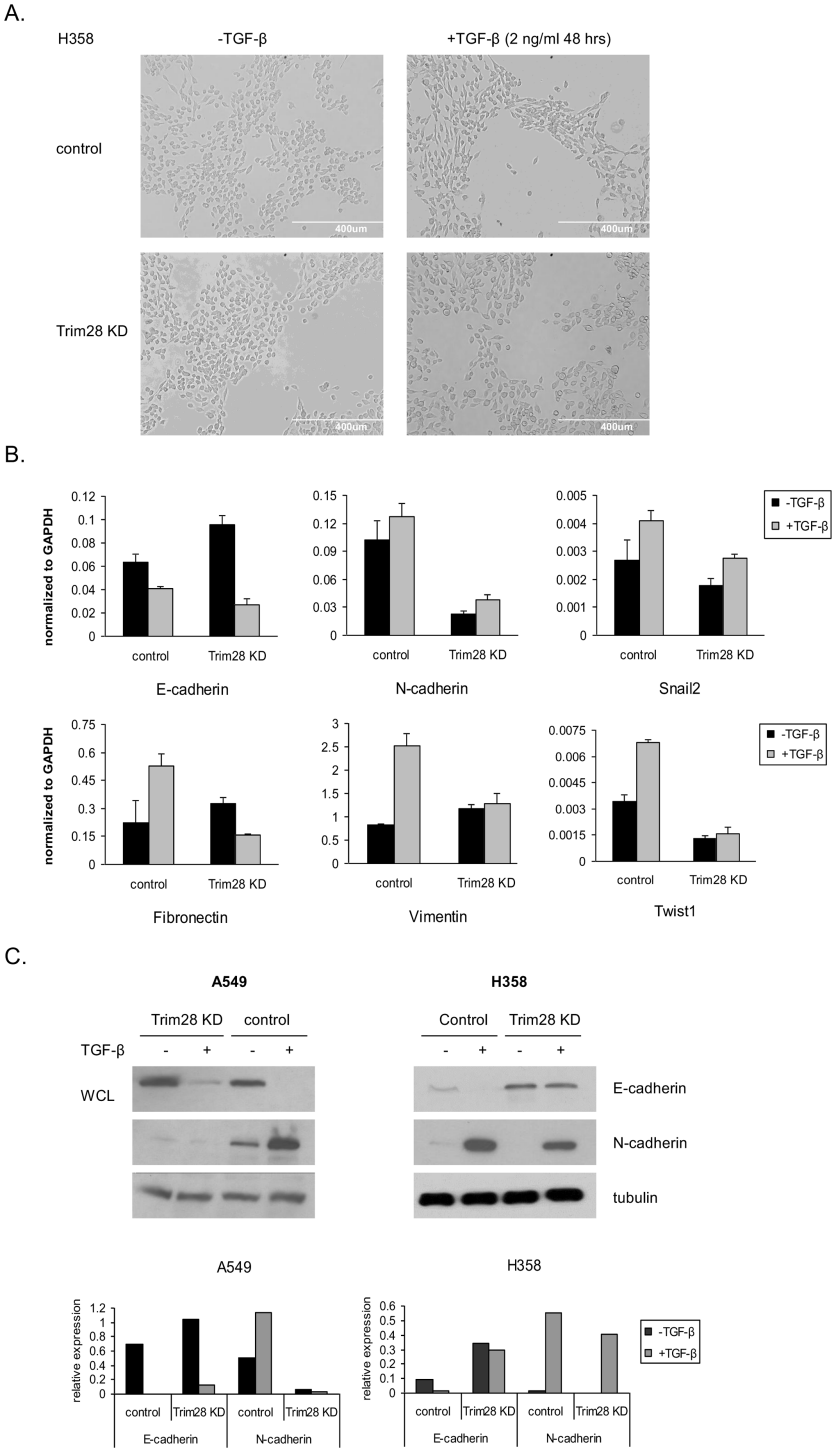
Trim28 is required for TGF-β-induced EMT. *A*, control and Trim28 knockdown cells were treated with TGF-β or left untreated. Cells were photographed at 40× magnification. *B*, RNA was extracted from control and Trim28 knockdown A549 cells treated with TGF-β as above or left untreated. Real-time PCR was performed and the expression of target genes was determined. *C*, control and Trim28 knockdown A549 and H358 cells were treated with TGF-β (2 ng/ml) until morphology changes were observed or left untreated. Whole cell lysates were subjected to western blotting using antibodies as indicated.

TGF-β treated cells will undergo EMT which can be characterized by morphological cell changes from pebble-like to spindle-like shape [32]. To determine whether Trim28 is required for TGF-β-induced EMT, we treated control and Trim28 knockdown cells with TGF-β (2 ng/ml) and observed the morphological change over time. Fig. 4A demonstrates that the acquisition of mesenchymal morphology in control H358 induced by TGF-β was not observed in Trim28 knockdown cells under the same condition strongly suggesting that Trim28 is involved in TGF-β-induced EMT. Using real-time PCR, we examined the mRNA level of E-cadherin and other known mesenchymal markers in A549 control and Trim KD cells after TGF-β treatment. Although in Trim28 knockdown cells some mesenchymal genes show slight up-regulation, the robust induction of mesenchymal markers N-cadherin, fibronectin, vimentin, and Twist1 caused by TGF-β treatment was significantly attenuated (Fig.4B). Western blot results (Fig.4C) confirm that TGF-β is able to reduce E-cadherin and up-regulate N-cadherin expression in control cells, but it fails to do so in Trim28 knockdown cells.

### Depletion of Trim28 reduces cell migration and invasion

**Figure 5:**
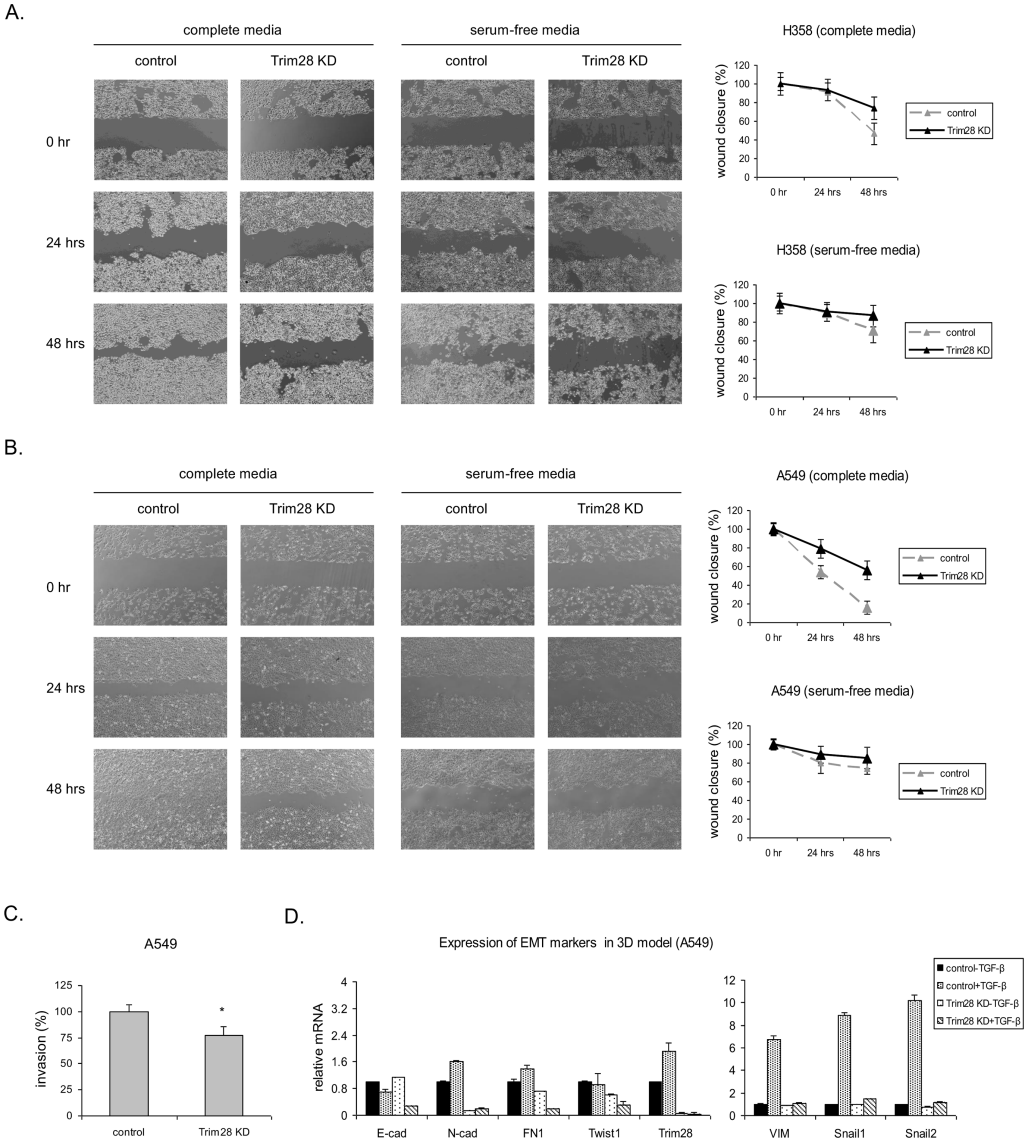
Trim28 knockdown attenuates cell migration. *A*, control and Trim28 knockdown H358 cells were subjected to wound-healing assay. Cells were photographed right after scratch, and 24 hrs and 48 hrs after scratch. *B*, control and Trim28 knockdown A549 cells were subjected to wound healing assay in the absence or presence of serum. Cells were photographed right after scratch and 24 hrs later. *C*, control and Trim28 knockdown cells were plated in triplicate on the top wells of Matrigel-coated or non-coated chambers. After 18 hours, cells that had invaded through the Matrigel layer were fixed and stained. The percentage of invasive cells is shown in the bar graph. *Asterisks* represent significant *p* values, *: *p*<0.05. *D*, control and Trim28 knockdown A549 cells were cultured in a three-dimensional model and treated with TGF-β (5 ng/ml) for 72 hrs. RNA was extracted from the cells and subjected to real-time PCR. The expression of target genes was determined.

The role of Trim28 in cell migration and invasion was evaluated by wound-healing assay and the Matrigel-based transwell invasion assay. In our previous studies, we found that Trim28 affects cell proliferation in A549 cells, but not significantly in H358 cells (unpublished data and [9]). Herein, we used culture media with or without FBS to exclude the contribution of cell proliferation in the determination of migration of A549 cells. Trim28 knockdown cells showed less wound closure than control cells (Fig.5A and 5B) with or without serum. In addition, the Matrigel-based transwell invasion assay showed that Trim28 knockdown inhibited cell invasion of A549 cells (Fig.5C).

Three-dimensional cell cultures have been shown to undergo EMT more efficiently than monolayer cells [28]. To examine whether Trim28 knockdown represses EMT in a three-dimensional model, we seeded control and Trim28 knockdown A549 cells on the lids of p100 culture dish and allowed them to form hanging drops as described in *Materials and Methods*. After 72 hrs in the presence of TGF-β, cells were harvested and RNA extracted for real-time PCR analysis of EMT markers. Fig. 4B reveals that after TGF-β treatment the expression of Vimentin, Snail1, and Snail2 in control A549 cells changed more dramatically (6 to 10 fold) in the 3D model (Fig. 5D) than those in monolayers (1.5 to 2.5 fold). However, these EMT markers were not up-regulated in Trim28 knockdown cells after TGF-β treatment. Together, these results support our finding that Trim28 is a regulator of EMT.

### Trim28 alters histone 3 modifications on E-cadherin and N-cadherin promoters

**Figure 6:**
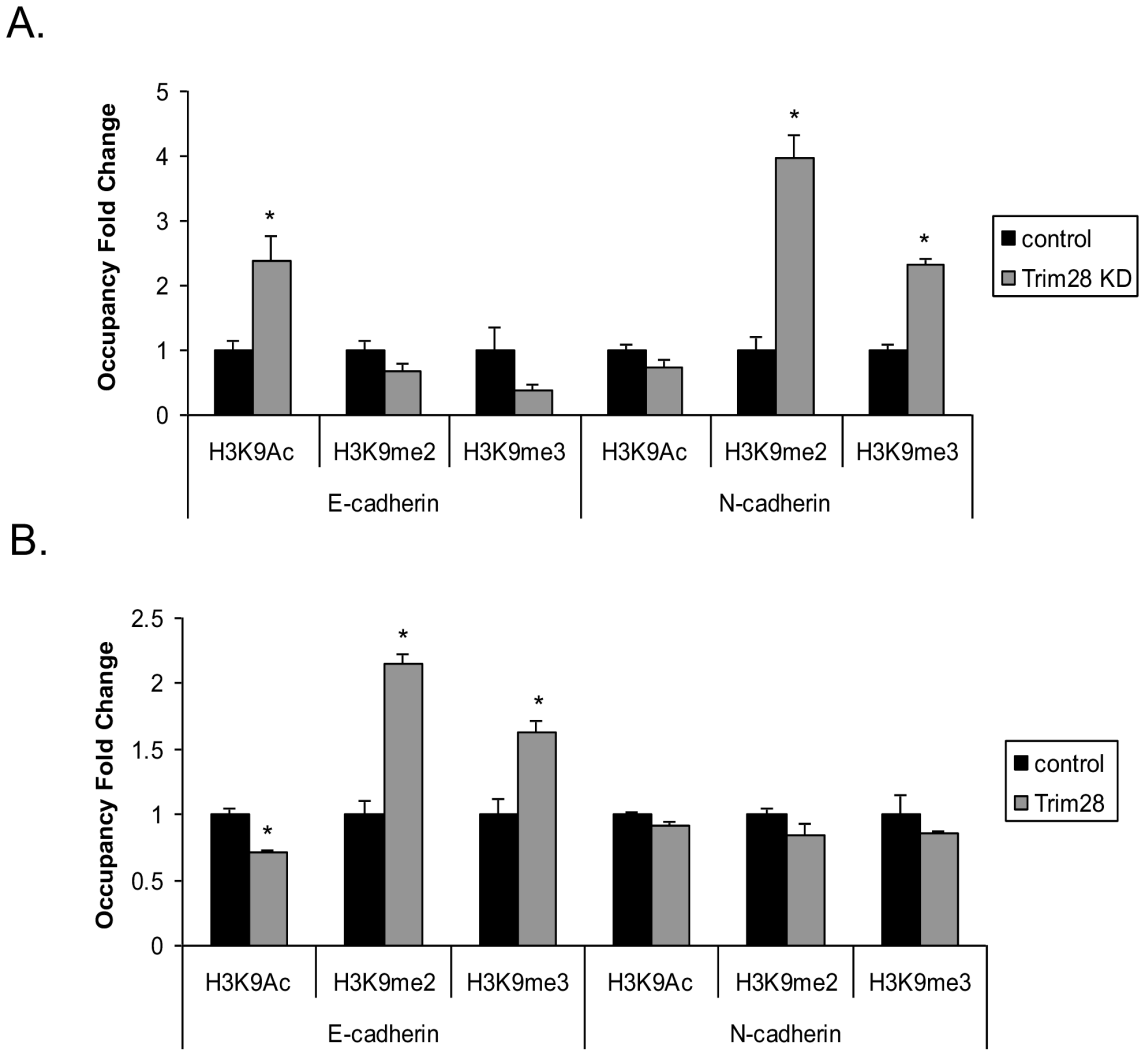
Trim28 regulates histone modifications of E-cadherin and N-cadherin promoters. *A*, ChIP assays were conducted in control and Trim28 knockdown A549 cells. Quantitative PCR was performed to examine the occupancy of IgG, acetylated histone 3 K9, dimethylated histone 3 K9, and trimethylated histone 3 K9 on E-cadherin and N-cadherin promoters. B, ChIP assays were conducted in control and Trim28 stably expressed A549 cells. Quantitative PCR was performed to examine the occupancy of IgG, acetylated histone 3 K9, dimethylated histone 3 K9, and trimethylated histone 3 K9 on E-cadherin and N-cadherin promoters.

Trim28 has been demonstrated to regulate gene expression through modification of histones of target gene promoters [8]. Known partners of Trim28 include SETDB1 [19], a histone 3 lysine 9-specific methyltransferase and histone deacetylases such as HDAC1 [8]. In our previous work, we found that Trim28 knockdown increases histone 3 lysine 9 acetylation on certain target promoters [9]. Herein, we explored whether Trim28 affects histone 3 modification of the E-cadherin and N-cadherin promoters. ChIP assays were employed and IgG, acetyl-H3K9, dimethyl-H3K9, and trimethyl-H3K9 antibodies were used. As shown in Fig. 6A, in Trim28 deficient cells, acetylation is increased whereas the methylation is decreased on histone 3 lysine 9 of E-cadherin promoter compared to control shRNA cells. An opposite effect on N-cadherin was observed in the same cells.

In addition, to demonstrate whether Trim28 overexpression can affect the E-cadherin and N-cadherin promoters, we used pcDNA3 control and Trim28-proficient A549 cells and performed ChIP assay. In Fig. 6B, contrary to the results observed in Trim28 knockdown cells, the acetylation of histone H3K9 is decreased and methylation is increased in Trim28-proficient cells. On N-cadherin promoter, the acetylation of histone 3 H3K9 is increased and methylation is decreased. Altogether these results indicate that Trim28 alters E-cadherin and N-cadherin expression (at least in part) by histone modification that in turn regulate their transcription.

## Discussion

We have shown that Trim28 can affect the post-translational modification of histone 3 on E-cadherin and N-cadherin promoters. At this point we have not elucidated the specific mechanisms involved. Trim28 may mediate these modifications directly or indirectly. The direct mechanism would involve Trim28 and Trim28-associated histone modifiers being recruited to cadherin promoters by EMT-regulating transcription factors. Trim28 has been shown to bind and regulate many transcription factors in this fashion [16], [19], [33]. However, we have designed primers targeting the -2 kb to +0.5 kb of E-cadherin and N-cadherin promoters and failed to detect a Trim28 binding region by ChIP assay (unpublished observations). Another possibility is that Trim28 regulates the expression of EMT-related transcription factors which will further regulate EMT genes. The transcription factors that may reside directly on E-cadherin promoter include Snail1, Snail2, ZEB1, ZEB2, Twist1 and FOXC2 [4]. In our study, we found that the expression of many transcription factors is changed with Trim28 overexpression or deficiency which provides evidence to support the possibility. Further investigations will explore this mechanism.

**Figure 7:**
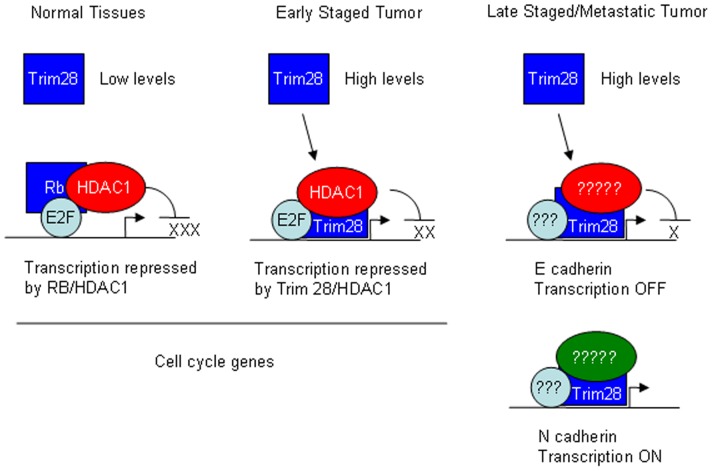
Hypothetical model to explain the role of Trim28 in the stages of lung adenocarcinoma. This model explains this work and our previous work demonstrating a tumor suppressor role for Trim28 [9].

The most notable component of our work described here is that Trim28 influences TGF-β-induced EMT in lung cancer through transcriptional regulation of multiple epithelial and mesenchymal markers. Mouse conditional knockout studies have clearly indicated that Trim28 and related family member can behave as tumor suppressors [34]; however, other studies indicate that Trim28 has elevated levels and oncogenic effects in a variety of cancers [21], [35]. We propose the model shown in Fig. 7 to explain the dual role of Trim28 in lung cancer development and metastasis. We hypothesize that Trim28, through regulation of multiple alternative pathways may have both tumor suppressing and oncogenic activities much like the TGF-β signaling pathway is known to play a complex role tumorigenesis. On one hand, TGF-β1 negatively regulates cell cycle and serves as a tumor suppressor in normal and pre-malignment tissues. On the other hand, TGF-β, secreted by tumor cells, promotes tumor invasion and metastasis [36]. We propose that Trim28 contributes significantly to TGF-β's ability to restrain cell growth and for TGF-β1 to induce EMT. These possibilities are not mutually exclusive and this network can be even more complex when the stages and types of cancer are considered. As shown in Figure 7, in normal tissues and early stage cancers, Trim28 is responsible for cell-cycle regulation through E2F transcription factors and HDACs; therefore, Trim28 shows an anti-proliferative function and acts as a tumor suppressor. In late stage or metastatic cancers, high level of Trim28 contribute to EMT through transcription regulation of epithelial and mesenchymal genes, as a result, cells with high level of Trim28 tend to have a more invasive and metastatic nature. This model is supported by reports in the literature [13], [24] and our findings [9]. Our results may shed light toward understanding the dual roles of TGF-β signaling in tumors.

## Supporting Information

Figure S1 **Trim28 deficiency reduces the expression of N -cadherin.** *C*ontrol and Trim28 knockdown A549 cells were stained (as described under “Experimental Procedures”) and examined using confocal immunofluorescence microscopy, as follows: N-cadherin (green, top panel), β-tubulin (green, bottom panel), and DAPI (blue). Merged images are at the bottom.
(TIF)

Figure S2 **Trim28 expression show negative correlation with E cadherin expression and positive correlation with N-cadherin expression in a series of non small cell lung cancer lines.** A, whole cell lysates were subjected to western blotting using the indicated antibodies. B, band intensities were quantified and plotted on a log scale. Correlations are not statistically significant.
(TIF)
