# Supplementary figures and images for: Trim28 Contributes to EMT via Regulation of E-Cadherin and N-Cadherin in Lung Cancer Cell Lines
Source: PLoS One. 2014 Jul 1;9(7):e101040. doi: 10.1371/journal.pone.0101040 (PMC4077753; doi:10.1371/journal.pone.0101040)

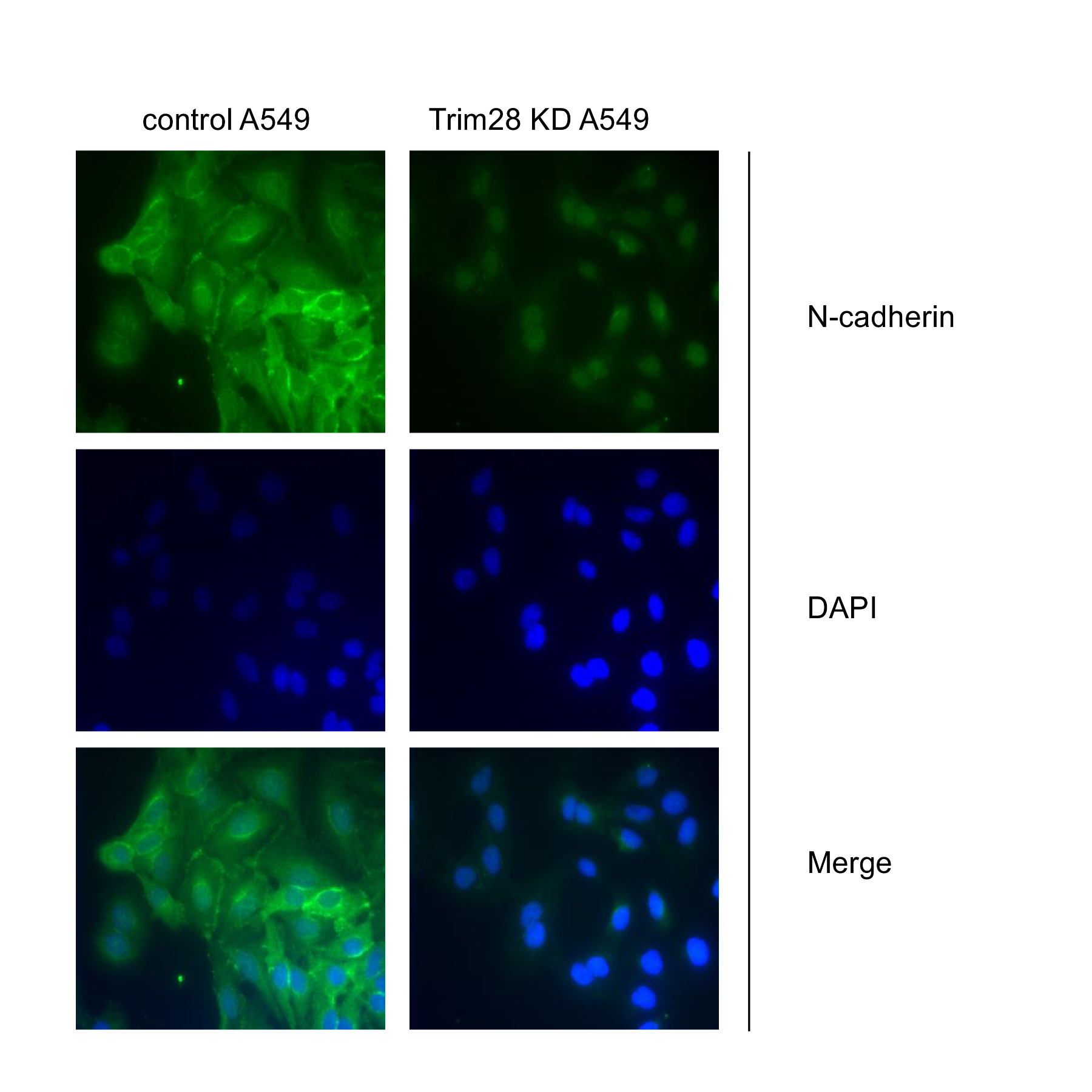

Supplement: Figure S1 — Trim28 deficiency reduces the expression of N -cadherin. Control and Trim28 knockdown A549 cells were stained (as described under “Experimental Procedures”) and examined using confocal immunofluorescence microscopy, as follows: N-cadherin (green, top panel), β-tubulin (green, bottom panel), and DAPI (blue). Merged images are at the bottom. (TIF) [file pone.0101040.s001.tif]

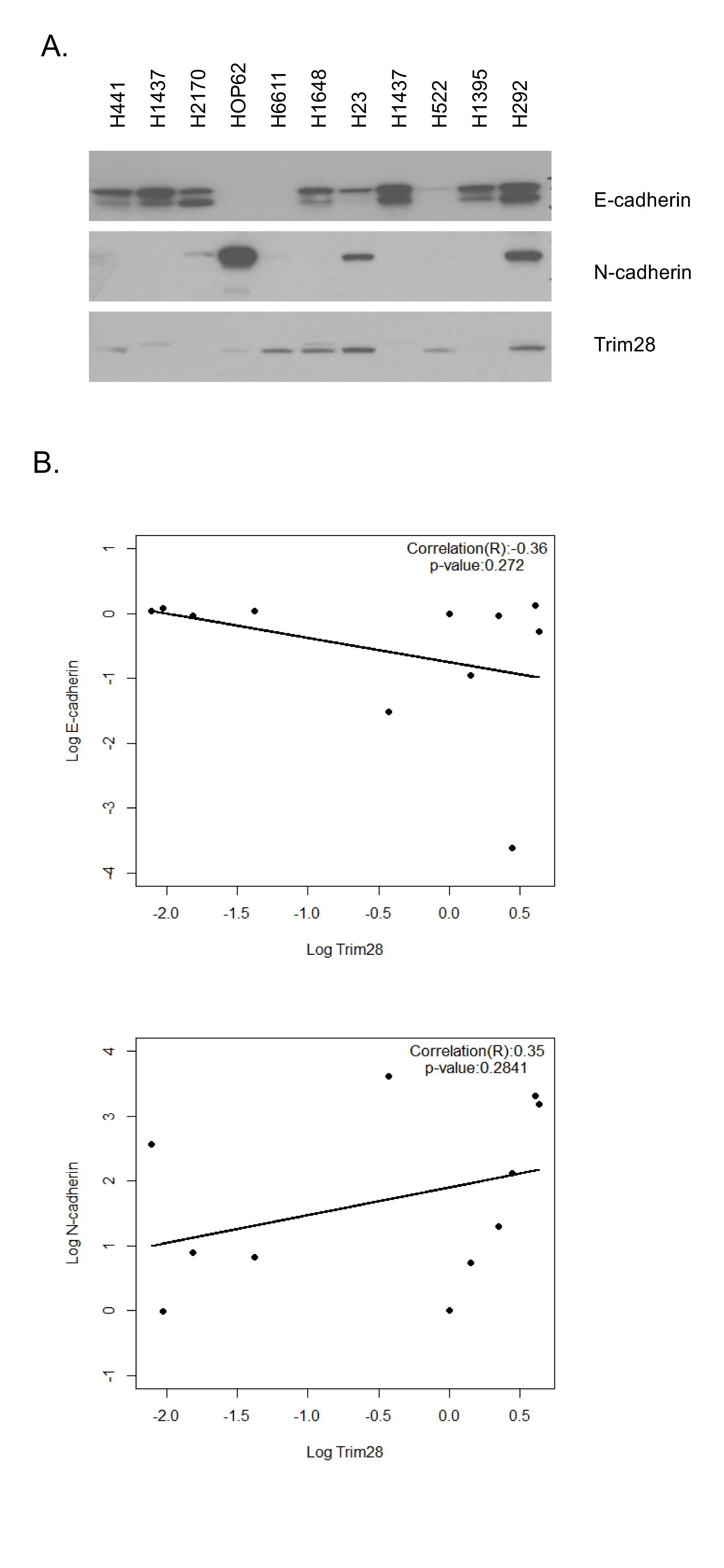

Supplement: Figure S2 — Trim28 expression show negative correlation with E cadherin expression and positive correlation with N-cadherin expression in a series of non small cell lung cancer lines. A, whole cell lysates were subjected to western blotting using the indicated antibodies. B, band intensities were quantified and plotted on a log scale. Correlations are not statistically significant. (TIF) [file pone.0101040.s002.tif]
